# Immunomodulatory, trypanocide, and antioxidant properties of essential oil fractions of *Lippia alba* (Verbenaceae)

**DOI:** 10.1186/s12906-021-03347-6

**Published:** 2021-07-02

**Authors:** Wendy Lorena Quintero, Erika Marcela Moreno, Sandra Milena Leal Pinto, Sandra Milena Sanabria, Elena Stashenko, Liliana Torcoroma García

**Affiliations:** 1 Infectious Disease Research Program, Universidad de Santander, Bucaramanga, Santander Colombia 680006; 2 Fundación Cardiovascular de Colombia, Floridablanca, Santander Colombia 681004; 3 National Research Center for the Agroindustrialization of Aromatic and Medicinal Tropical Species (CENIVAM), Universidad Industrial de Santander, Bucaramanga, Colombia 680002

**Keywords:** Chagas disease, *Trypanosoma cruzi*, Immunomodulation, Antioxidant, *Lippia alba*, Essential oil fractions

## Abstract

**Background:**

Parasite persistence, exacerbated and sustained immune response, and continuous oxidative stress have been described to contribute to the development of the cardiac manifestations in Chronic Chagas Disease. Nevertheless, there are no efficient therapies to resolve the *Trypanosoma cruzi* infection and prevent the disease progression. Interestingly, trypanocide, antioxidant, and immunodulatory properties have been reported separately for some major terpenes, as citral (neral plus geranial), limonene, and caryophyllene oxide, presents in essential oils (EO) extracted from two chemotypes (Citral and Carvone) of *Lippia alba*. The aim of this study was to obtain *L. alba* essential oil fractions enriched with the aforementioned bioactive terpenes and to evaluate the impact of these therapies on trypanocide, oxidative stress, mitochondrial bioenergetics, genotoxicity, and inflammatory markers on *T. cruzi-*infected macrophages*.*

**Methods:**

*T. cruzi*-infected J774A.1 macrophage were treated with limonene-enriched (ACT1) and citral/caryophyllene oxide-enriched (ACT2) essential oils fractions derived from Carvone and Citral-*L. alba* chemotypes, respectively.

**Results:**

ACT1 (IC_50_ = 45 ± 1.7 μg/mL) and ACT2 (IC_50_ = 80 ± 1.9 μg/mL) exhibit similar trypanocidal effects to Benznidazole (BZN) (IC_50_ = 48 ± 2.5 μg/mL), against amastigotes. Synergistic antiparasitic activity was observed when ACT1 was combined with BZN (∑FIC = 0.52 ± 0.13 μg/mL) or ACT2 (∑FIC = 0.46 ± 1.7 μg/mL). ACT1 also decreased the oxidative stress, mitochondrial metabolism, and genotoxicity of the therapies. The ACT1 + ACT2 and ACT1 + BZN experimental treatments reduced the pro-inflammatory cytokines (IFN-γ, IL-2, and TNF-α) and increased the anti-inflammatory cytokines (IL-4 and IL-10).

**Conclusion:**

Due to its highly trypanocidal and immunomodulatory properties, ACT1 (whether alone or in combination with BZN or ACT2) represents a promising *L. alba* essential oil fraction for further studies in drug development towards the Chagas disease control.

**Supplementary Information:**

The online version contains supplementary material available at 10.1186/s12906-021-03347-6.

## Background

Chagas disease is a serious parasitic infection caused by the protozoan *Trypanosoma cruzi* (Chagas, Kinetoplastida: tripanosomatidae), and presently constitutes the third-greatest parasitic disease burden worldwide, following malaria and schistosomiasis [1], with an estimated cost of USD $7.19 billion per year and USD $188.80 billion per lifetime [2]. In Colombia, its prevalence is estimated at 0.7–1.2 million cases, with more than 8 million more at risk [3].

In a vertebrate host, *T. cruzi* can invade any type of nucleated cell and, once the parasite is internalized, an acute or subacute infection is established. During the cellular immune response, resident macrophages are activated, and Reactive Oxygen Species (ROS) are produced; causing a respiratory burst that serves as the host’s first line of defense against the parasite. Similarly, in the chronic phase of *T. cruzi* infection, there is a significant increase in oxidative stress, as evidenced by the diminished activity of antioxidant enzymes such as manganese-dependent superoxide dismutase (Mn-SOD) and glutathione peroxidase (GPx), and the instability of reduced glutathione (GSH) [4].

These ROS modulate the cytokine responses (IL-4, IL- 1β, IL-6, TNF-α, and IFN-γ) and the proliferation of inflammatory cells, predominantly CD4^+^ and CD8^+^ T lymphocytes [5]. In general, this process represents a successful parasitemia control strategy, given that about 70% of hosts may remain asymptomatic for their entire life, even with a persistent *T. cruzi* presence in their tissues. However, in the remaining cases, chronic manifestations of Chagas disease may be developed due to the combination of several elements, such as parasite persistence, exacerbated and sustained immune response, and continuous oxidative stress, among others [5]. This stage is characterized by massive cardiac fibrosis and progressive decline of ventricular contractile function, which may eventually result in organ failure and death [6].

Accordingly, chronic chagasic patients exhibit a higher profile of Th1-type cytokines with suppression of Th2-type cytokines, producing more IFN- *γ* and less IL-10 in comparison to patients in the indeterminate phase. This dysregulation of the Th1/Th2 response has been associated with an inefficient cardiac function, and therefore with a worse prognosis [7]. Additionally, some specific chemokines are produced in tissues in response to infection, and are crucial in defining the leukocyte subtypes that will make up the inflammatory infiltrate in the hearts of infected organisms. Among these chemokines, IFN-γ, for example, activates macrophages and cardiomyocytes infected with *T. cruzi* to produce TNF-α, nitric oxide (NO), ROS, and other Reactive Nitrogen Species (RNS). This process promotes high trypanocidal activity and is also associated with a considerable degree of damage to the affected tissue [5].

Unfortunately, Chagas disease, has drawn little interest from the pharmaceutical industry, largely due to economic considerations. Currently, the only medicines that exhibit robust evidence for their effective treatment of Chagas disease are Benznidazole (BZN) and Nifurtimox (NFX), discovered more than five decades ago, which are provided as a mandatory therapy in cases of confirmed acute infection [6, 8]. When applied during the disease’s acute phase, these medications have demonstrated cure rates of up to 80%; however, there is no clear evidence to indicate their efficacy in other stages of the disease [6, 8].

During the past decades, multicentric studies have been conducted using a variety of treatment schemes to clarify the future applicability of BZN and NFX [6, 8]. Similarly, efforts have been undertaken to explore alternative and more holistic therapies for the disease. With this respect, the goal of this work was to evaluate, in an in vitro model, therapies that addressed not only to control the parasite load, but also to modulate the immune and oxidative responses in the *T. cruzi*-infection. In this regard, essential oils from aromatic plants represent an important source of bioactive compounds, generally with low toxicity and valuable potential as broad-spectrum antimicrobial agents. Some examples of essential oils with trypanocidal activity include plants as *Ambrosia* sp., *Syzygium aromaticum, Eugenia caryophyllata, Cymbopogon citratus, Artemisia absinthuim, Zanthoxylum pistaciifolium, Aniba rosaeodora,* and *Lippia alba* [9, 10]*.* Essential oils whose major compounds are limonene, citral (neral plus geranial), and caryophyllene oxide (OCN) have been shown to effectively inhibit the growth of *T. cruzi* [10, 11]. Relevant immunomodulatory and anti-oxidant effects have also described individually for these terpenes in in vitro and in vivo mammalian models [11, 12]. The aformentioned terpenes exhibited the ability to impair the expression of pro-inflammatory cytokines (such as IFN-γ and IL-2), while increasing anti-inflammatory interleukines like IL-4 and IL-10 [11, 12]. Interestingly, limonene, citral, and OCN are found in significant percentages in essential oils extracted from Carvone and Citral chemotypes of *Lippia alba* (Mill.) N.E. Brown (Verbenaceae), a aromatic shrub that grow wild in Colombia [10]. The main aim of the present study was to explore in vitro*,* a potential therapy based on fractions isolated from *L. alba* essential oils, more effective and safer against the *T. cruzi*-infection, which also to be able to contribute in modulating genotoxicity, pro-inflammatory immune response (IFN-γ, TNF-α, RANTES, and IL-2), and oxidative stress (ROS) response.

## Methods

### Plant material

For this work, only fresh and matures leaves of Citral and Carvone chemotypes specimens of *L. alba* (Mill.) N.E. Brown were harvested. This plant is a shrub that bloom all year. The specimens were grown under standard conditions [10], at the National Research Center for the Agroindustrialization of Aromatic and Medicinal Tropical Species (Universidad Industrial de Santander), in Bucaramanga, Colombia, at an altitude of 960 m above mean sea level, in rainy season (April to November, mean temperature of 24.5 °C, relative humidity of 80–90% and 3–5 mm of daily precipitation). Plant collecting permit was obtained from the Ministerio de Ambiente y Desarrollo Sostenible (Colombia), under Resolution 1761 of 1st November 2019. The exsiccate with their voucher numbers were deposited at the Colombian National Herbarium (Universidad Nacional de Colombia) under Herbarium Codes COL480750 and COL512077, for Carvone and Citral chemotypes of *L. alba*, correspondingly.

### Essential oils and fractions isolation

The essential oils extraction was performed through steam distillation in a 0.4 m^3^ stainless-steel still, from 80 kg of freshly collected plant material (fresh and mature leaves). The separation of oils was made by decantation, dried with anhydrous sodium sulphate, and stored in amber flasks at 4 °C. Reduced-pressure fractional distillation of the essential oils was carried out in a B/R Instruments (Easton, Maryland, USA) 800 High Efficiency Micro Distillation device. The ACT1 fraction (limonene-enriched) was collected at 67 °C, during the *L. alba* Carvone-chemotype essential oil distillation process at 12 Torr. ACT2 was collected at temperatures of 115 °C through *L. alba* Citral-chemotype essential oil distillation process at 7 Torr. Fractions were stored in dark glass bottles at 4 °C, until analysis.

### GC and GC-MS analysis

The composition of the EO fractions was analyzed using an Agilent Technologies (AT) 7890 gas chromatograph equipped with an AT 5975C mass-selective detector (electron ionization, 70 eV). For the tentative identification of essential oil components, retention indices are used. In modern GC, where the oven temperature can be programmed, and can be changed linearly, the next equation is employed for the calculation of retention indices in which logarithms of retention times (tR), according to van den Dool and Kratz aren’t used [13]. Instead, the absolute values of tR appear in the equation, as follows:

$$ LRI=100n+100\ \left(\frac{tRx- tRn}{tRN- tRn}\right) $$

These thus obtained indices are called linear retention indices (LRI). Identification of EO fraction components was accomplished based on comparison of LRI on both non-polar (DB-5MS, 60 m × 0.25 mm × 0.25 μm) and polar (DB-WAX, 60 m × 0.25 mm × 0.25 μm) columns (J & W Scientific, Folsom, CA, USA), with those of authentic standards (geranial, geraniol, neral, nerol, carvone, limonene, *trans*-β-caryophyllene) and by comparison of their mass spectral fragmentation patterns with those of the scientific literature and databases (Wiley-2008, NIST-2017, QUADLIB-2007) (Biomolecular Measurement Division, Gaithersburg, Maryland, USA). An AT 6890 gas chromatograph with flame ionization detector was used for component quantification. Injection volume was 1 μL, each sample was injected three times and the mean of three relative GC areas was reported for each compound. Both chromatographs used helium as carrier gas (1 mL/min) and their oven temperatures were programmed from 50 °C to 250 °C at 4 °C/min. The mixture of *n*-paraffines C_6_-C_25_ was purchased from AccuStandard Inc. (New Haven, CT, USA) and used for linear retention indices calculations.

The stock solutions of the fractions were prepared in dimethylsulfoxide (DMSO) and stored at a temperature of 4 °C, protected from light. The working solutions were diluted in Dulbecco’s Modified Eagle Medium (D-MEM) culture medium until the desired concentrations were obtained, targetting a final DMSO concentration of < 0.1%.

### Reference drugs

The reference drug, Benznidazole (Radanil®, Roche), was donated by the Secretary of Health from the Department of Santander and purified at the Universidad Santo Tomás de Aquino (Bucaramanga).

### Cell cultures

Macrophages from the J774A.1 (ATCC TIB-67) cell line donated by the Cellular and Functional Biology and Biomolecular Engineering Group from the Universidad Antonio Nariño, Colombia, were cultured and maintained in D-MEM medium (Life Technology, California, USA) supplemented with 1% penicillin-streptomycin and 10% inactivated Fetal Bovine Serum (iFBS) (Life Technology, California, USA) at 37 °C, 5% CO_2_ and 95% humidity. Epimastigote forms of *T. cruzi* I (TcI) SYLVIO X-10 strain, donated by the Fundación Cardiovascular de Colombia (FCV), were grown in Liver Infusion Tryptose (LIT) medium (Merck, Darmstadt, Germany) supplemented with 10% iFBS at 28 °C, until reaching a stationary growth phase after 15 days. The trypomastigotes were obtained by infection of a confluent monolayer of J774A.1 cells with 15 day-old stationary growth phase epimastigotes and incubated, under the same condition described above for J774A.1 cells [10].

### Cytotoxicity in J774A.1 murine macrophages

For cytotoxicity tests, J774A.1 macrophages (1500 cells/well) were incubated in 96-well flat-bottom plates at 37 °C, 5% CO_2_, and 95% humidity for 24 h until monolayer formation. Subsequently, these macrophages were exposed to varying concentrations of enriched fractions (600, 300, 150, and 75 μg/mL), for 24 h, after which 10 μL/well of the cell proliferation reagent WST-1 (Roche, Mannheim, Germany) was added. Absorbance was measured after 4 h of incubation at 450 nm using an iMark™ Microplate Absorbance Reader (BioRad, Madrid, Spain). The cytotoxicity percentage was calculated using [(OD_450nm_ control–OD_450nm_ treatment)/OD_450nm_ treatment)] × 100, and results were expressed as Cytotoxic Concentration 50 (CC_50_). Untreated or BZN-treated cells were used as negative and positive controls, respectively [6]. All analyses were performed in triplicate in three independent experiments.

### Antiparasitic activity in intracellular amastigotes of *T. cruzi*

To evaluate the trypanocidal activity of the enriched fractions, J774A.1 macrophages (1500 cells/well), incubated in 16-well plates, were infected with tripomastigotes in a 1:5 ratio (cell:parasite) for 24 h or until the development of intracellular amastigotes. Infected cells were maintained under the same culture conditions described above and treated with varying concentrations of enriched fractions for 24 h. The negative control was represented by infected and untreated macrophages; while infected and BZN-treated cells were used as a reference (positive control). To assess growth inhibition, the plates were fixed with methanol and stained with Wright (Merck, Darmstadt, Germany). The percentage of infected macrophages in a total of 300 cells was determined by light microscopy [10]. The results were expressed as Inhibitory Concentration 50 (IC_50_).

### Pharmacological interactions in J774A.1 murine macrophages

Pharmacological interactions between ACT1 + ACT2 and ACT1 + BZN were tested in both uninfected and *T. cruzi*-infected (amastigote) macrophages, using the fixed-ratio isobologram method described by Fivelman [14]. The individual CC_50_ and IC_50_ data calculations for the enriched fraction and BZN were used as a base, and the pharmacological interactions were assessed under the combinations described in Table 1.

**Table 1:** Matrix of pharmacological interactions tested on amastigotes of *Trypanosoma cruzi* or J774A.1 macrophage cell

Mixture	Factorial combinations IC_50_ or CC_50_ Values			Equivalent Compound Concentration		
	ACT1	ACT2	BZN	ACT1 (μg/mL)	ACT2 (μg/mL)	BZN (μg/mL)
1	0	8X IC_50_/CC_50_		–	640/1704	
2	½X IC_50_/CC_50_	4X IC_50_/CC_50_		22.5/229	320/852	
3	1X IC_50_/CC_50_	2X IC_50_/CC_50_		45/458	160/426	
4	2X IC_50_/CC_50_	1X IC_50_/CC_50_		90/916	80/213	
5	4X IC_50_/CC_50_	½X IC_50_/CC_50_		180/1832	40/106.5	
6	8X IC_50_/CC_50_	0		360/3664	–	
7	0		8X IC_50_/CC_50_	–		384/2388
8	½X IC_50−_CC_50_		4X IC_50_/CC_50_	22.5/229		192/1194
9	1X IC_50_/CC_50_		2X IC_50_/CC_50_	45/458		96/597
10	2X IC_50_/CC_50_		1X IC_50_/CC_50_	90/916		48/298.5
11	4X IC_50_/CC_50_		½X IC_50_/CC_50_	180/1832		24/149.3
12	8X IC_50_/CC_50_		0	360/3664		–

*ACT1* limonene-enriched fraction from Carvone-chemotype of *Lippia alba*, *ACT2* citral/caryophyllene oxide enriched fraction from Citral-chemotype of *Lippia alba*, *BZN* Benznidazole, *X* Multiplier, *IC*_*50*_ Inhibitory Concentration 50, *CC*_*50*_ Cytotoxic Concentration 50

Fractional Inhibitory Concentration (FIC) was calculated in order to evaluate susceptibility, using the formula FIC = (compound X_CI50_ in combination)/(compound Y_CI50_ alone). The sum of FIC (ΣFIC) was defined as: ΣFIC = compound X_FIC_ + compound Y_FIC._ In this manner, synergistic: $\overline{\mathrm{X}}$ΣFIC< 1; antagonistic: $\overline{\mathrm{X}}$ΣFIC> 1; and additive: $\overline{\mathrm{X}}$ΣFIC = 1 interactions were defined [15].

### Genomic damage to J774A.1 murine macrophages

Macrophages treated for 24 h with CC_50BZN_ (positive control) and the three best pharmacological interactions of each combination (ACT1 + ACT2 and ACT1 + BZN) (Table 2) were centrifuged, and the pellet subsequently mixed with low-melting-point agarose at 0.5% and added to slides previously treated with 1.5% agarose. Once the agarose had solidified, the slides were immersed in lysis buffer for 2 h, after which they were washed with alkaline buffer in an electrophoresis chamber for 20 min, with the electrophoretic run carried out at 25 V for 40 min. Finally, the slides were immersed in neutralization buffer, fixed with methanol, and stained with DAPI (1 μg/mL, Sigma Aldrich) to visualize the damage caused to DNA by bioactive enriched fractions (fluorescence microscopy, Nikon Eclipse Ni, Tokyo, Japan). DNA migration as an indicator of damage to the genome was determined by visual scoring, taking into account the length of the comet’s tail in accordance with Collins [16]. The damage was scored on a 5-point scale: from 0 (no damage evident) up to 4 (higher damage) [16].

**Table 2:** Combinations tested in genotoxicity assay on J774A.1 macrophage cell

Mixture	Factorial combinations in CC_50_ Values			Compounds concentration		
	ACT1	ACT2	BNZ	ACT1 (μg/mL)	ACT2 (μg/mL)	BNZ (μg/mL)
3	1X CC_50_	2X CC_50_		458	426	–
4	2X CC_50_	1X CC_50_		916	213	–
5	4X CC_50_	½X CC_50_		1832	106.5	–
10	2X CC_50_	–	1X CC_50_	916	–	298.5
11	4X CC_50_	–	½X CC_50_	1832	–	149.3
12	8X CC_50_	–	0	3664	–	–

*ACT1* limonene-enriched fraction from Carvone-chemotype of *Lippia alba*, *ACT2* citral/caryophyllene oxide enriched fraction from Citral-chemotype of *Lippia alba*, *BZN* Benznidazole, *X* Multiplier, *CC*_*50*_ Cytotoxic Concentration 50

### Morphological changes to J774A.1 macrophages

The cell death phenotype was analyzed by fluorescent microscopy using phase contrast (Nikon Eclipse Ni, Tokyo, Japan). Briefly, uninfected and *T. cruzi-*infected cells treated with the best synergic mixtures (ACT1 + BZN and ACT1 + ACT2), were studied by DAPI (1 μg/m) and TUNEL assays (Molecular Probes, Invitrogen, CA, USA) for DNA fragmentation using a Terminal desoxynucleotidyl Transferase (TdT) label with d-UTP fluorescein. Evaluation of the mitochondrial membrane potential was assessed using 5 μM of MitoProbe™ JC-1 Assay (Molecular Probes, Invitrogen, CA, USA). Controls were provided by uninfected and untreated macrophage cultures (negative) and by cultures treated with a commercial kit solution (positive) in accordance with the manufacturer’ instructions.

### Oxidative stress on J774A.1 macrophages

Infected and uninfected J774A.1 macrophages were plated in 16-well microplates and treated with the best pharmacological interactions of ACT1 + ACT2 and ACT1 + BZN for 24 h, as previously mentioned. Subsequently, the culture medium was removed and rinsed three times with PBS before treatment with 4 μM of MitoSOX™ Red (Invitrogen, CA, USA), for 30 min at 37 °C, 5% CO_2_, and 97% humidity. Cells treated with the CC_50BZN_ were taken as a positive control. The cells were observed under a fluorescence microscope (Nikon Eclipse Ni, Tokyo, Japan) with emission/excitation wavelengths of 510/580 nm, respectively, and the fluorescence quantification was estimated with NIS-Element software (Tokyo, Japan) in accordance with the manufacturer’ instructions.

### Mitochondrial bioenergetics of J774A.1 macrophages both uninfected and infected with *T. cruzi*

Real-time changes in oxygen consumption rates (OCR, as a measure of oxidative phosphorylation) and extracellular acidification rates (ECAR, as a measure of lactate production) were examined using an XFe 24 extracellular flux analyzer (Seahorse Biosciencie, Billerica, United States). Briefly, J774A.1 macrophages infected with *T. cruzi* and treated with ACT1_CI25_ + ACT2_CI25_, ACT1_CI25_ + BZN_CI25_, and BZN_CI25_ were plated at 80,000 cells/well in 100 μL of media (D-MEM, 4 mM glutamine, 25 mM glucose, and 1 mM sodium pyruvate). Thereafter, 425 μL/well of additional media was added, and the J774A.1 cells were analyzed following the manufacturer’s instructions to obtain real-time measurements of OCR and ECAR baselines (in response to 1 μM oligomycin, 0.3 μM fluoro-carbonyl cyanide phenilhydrazone (FCCP), and 1 μM rotenone/antimycin A). Controls were respresented by uninfected and untreated macrophages and macrophages infected with *T. cruzi* without treatment [10].

### Cytokine assay

Cytokine was measured for each group (infected/uninfected macrophages, and those treated with the three best interactions between ACT1 + BZN and ACT1 + ACT2) using a “MILLIPLEX MAP Mouse Cytokine/Chemokine Magnetic Bead Panel” kit on the MAGPIX™ Luminex system (Texas, United States). Each sample was entered in two replicate wells and the kit was composed of four pro-inflammatory (IL-2, IFN-γ, TNF-α, and RANTES) and two anti-inflammatory cytokines (IL-4 and IL-10). The process was performed in acordance with the manufacturer’s instructions. Standards included all cytokines tested. Lipopolysaccharide (LPS) was used as pro-inflammatory control molecule for both uninfected and infected cell models.

### Statistical analysis

The statistical software Xlfit™ 4 (ID Business Solution, Guildford, England) was used to calculate CC_50_ and IC_50_ by sigmoidal regression. Cytotoxicity, pharmacological interactions, genotoxicity, and oxidative stress were tested in triplicate in three independent assays. Tests for normality (Shapiro Wilk) and homoscedasticity (Levene) were performed. Later, statistically-significant difference determinations were performed using the ANOVA test on SPSS 22.0 Software (IBM, Chicago, IL, USA). Multiple comparison analysis was accomplished using a Dunnett test with a 95% confidence level in all cases.

## Results

### Chemical composition of *Lippia alba* enriched fractions

In this study, two essential oils were extracted by steam distillation from 2 *L. alba* chemotypes (Citral and Carvone), which were fractioned by reduced-pressure fractional distillation for obtention of the two study fractions: ACT1, limonene-enriched fraction isolated from Carvone chemotype; and ACT2, citral/caryophyllene oxide-enriched fraction derived from Citral chemotype. Table 3 presents the relative chemical composition of the essential oils and their fractions, with their corresponding linear retention indices, obtained by mass spectra.

**Table 3:** Chemical composition of enriched fractions isolated from Citral and Carvone chemotypes of *L. alba*

Compound	LRI		Relative GC peak area, % (mean, *n* = 3)			
			Carvone-chemotype		Citral-chemotype	
	DB-5MS^a^	Lit^b^	EO	ACT1 [67 °C]	EO	ACT2 [115 °C]
6-Methyl-5-hepten-2-one	986	985	0.1	–	3.3	–
*p*-Cymene	1024	1024	0.1	–	1.1	–
Limonene	1030	1029	30.6	96	1.1	–
Terpinolene	1086	1087	0.3	–	0.5	–
Linalool	1098	1099	–	–	0.5	0.2
*trans*-Dihydrocarvone	1202	1201	0.1	–	–	–
Nerol	1228	1228	–	–	–	1.1
Neral	1240	1242	–	–	22.1	7.5
Carvone	1242	1242	51.2	1.4	–	–
Geraniol	1256	1254	–	–	5.6	6.9
Geranial	1270	1270	0.1	–	28.7	25.3
Piperitone	1342	1340	1.5	–	–	–
α-Copaene	1376	1376	0.4	–	0.5	–
β-Elemene	1390	1391	0.5	–	–	–
*trans*-β-Caryophyllene	1420	1420	0.3	–	12.2	18.3
α-Guaiene	1440	1439	–	–	1.9	2.9
β-Humulene	1454	1453	0.4	–	2.7	5.6
Germacrene D	1482	1480	0.3	–	2.6	4.9
β-Bisabolene	1508	1508	0.4	–	1.4	3.6
Caryophyllene oxide	1582	1580	0.2	–	2.3	7.6
Bicyclogermacrene	1496	1494	7.5	–	–	–

*EO* Essential oil, *ACT1* limonene-enriched fraction of Carvone-chemotype *Lippia alba* EO, *ACT2* citral/caryophyllene oxide-enriched fraction of Citral-chemotype *Lippia alba* EO, ^a^Linear retention index experimentally obtained on DB-5MS non-polar column; ^b^Linear retention indices from V.I. Babushok, et al. J. Phys.Chem. Ref.Data, Vol. 40, No. 4, 2011

### Trypanocidal activity of *L. alba* enriched fraction on intracellular amastigotes of *T. cruzi*

**Fig. 1:**
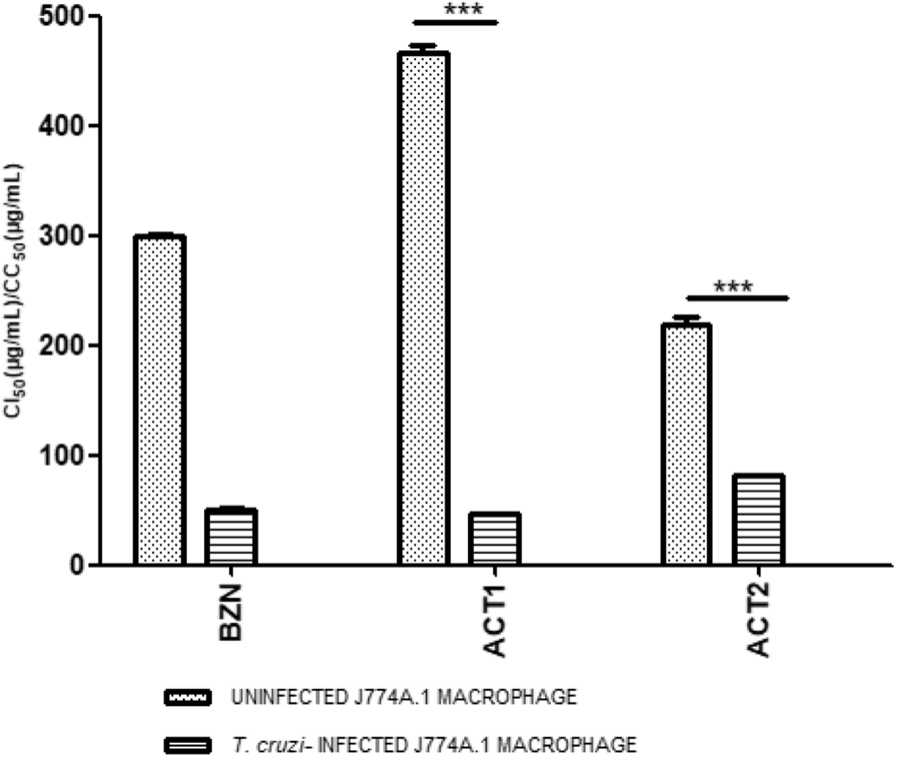
Cytotoxic and trypanocidal activity against amastigotes of *T. cruzi* by enriched fractions from *L. alba*. IC_50_: Inhibitory concentration 50; CC_50_: Cytotoxic concentration 50; ACT1: limonene-enriched fraction form Carvone-chemotype of *Lippia alba*; ACT2: citral/caryophyllene oxide enriched fraction from Citral-chemotype of *Lippia alba*

ACT1 exhibited the lowest IC_50_ on intracellular amastigotes (IC_50_ 45 ± 1.7 μg/ mL; SI = 10.1). This fraction also showed to be the more selective and less toxic treatment on uninfected J774A.1 macrophages (*p* < 0.0001), with a CC_50_ 1.5 times greater than that obtained by the reference medicine (ACT1: CC_50_ 458 ± 4.2 μg/mL; BZN: CC_50_ 298 ± 4.0 μg/mL) (Fig. 1). Conversely, ACT2 fraction was the least selective compound (IC_50_ 80 ± 1.9 μg/mL; CC_50_ 213 ± 1.2 μg/mL; IS = 2.6), although it also presented a significant trypanocidal effect (*p* = 0.009).

### Pharmacological interactions of enriched fraction on *T. cruzi* intracellular amastigotes

**Fig. 2:**
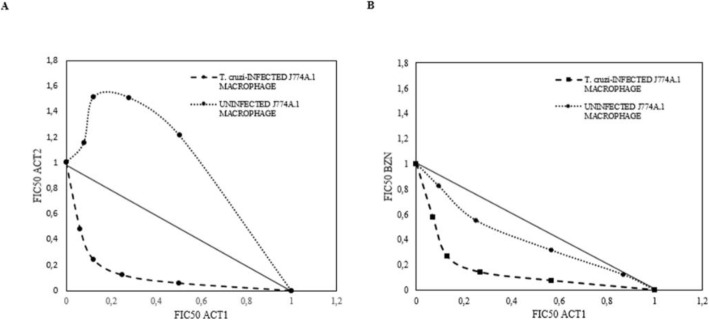
Isobolograms of the pharmacological interactions between ACT1 + ACT2 (**A**) and ACT1 + Benznidazole (**B**). The points above and below the line indicate an antagonistic and synergistic effect, respectively

Following the FIC value interpretation, the best trypanocidal interactions were showed by the combinations of ACT1 with ACT2. In this regard, the addition of ACT2, in the Mixture 3 (1X IC_50-ACT1_ + 2X IC_50-ACT2_), caused the 8.3-fold reduction in the IC_50_ of ACT1, upon intracellular amastigotes (∑FIC = 0.46) (Fig. 2a). Conversely, on J774A.1-uninfected macrophages, these fraction combinations presented an antagonistic effect (cytoprotection). Note that, Mixture 4 (2X IC_50-ACT1_ + 1X IC_50-ACT2_), presented a ∑FIC = 1.53, with a reduction of 1.72 and 2.5 times the ACT1_CC50_ and ACT2_CC50,_ respectively. Likewise, the combination of ACT1 with BZN, increased their cytotoxicity against intracellular amastigotes (∑FIC = 0.4, for the Mixture 10 (2X IC_50-ACT1_ + 1X IC_50-BZN_)), nevertheless, it also increased the toxic effect of the therapies, on uninfected macrophages (∑CIF =0.90) (Fig. 2b).

### Genotoxicity on J774A.1 macrophages

**Fig. 3:**
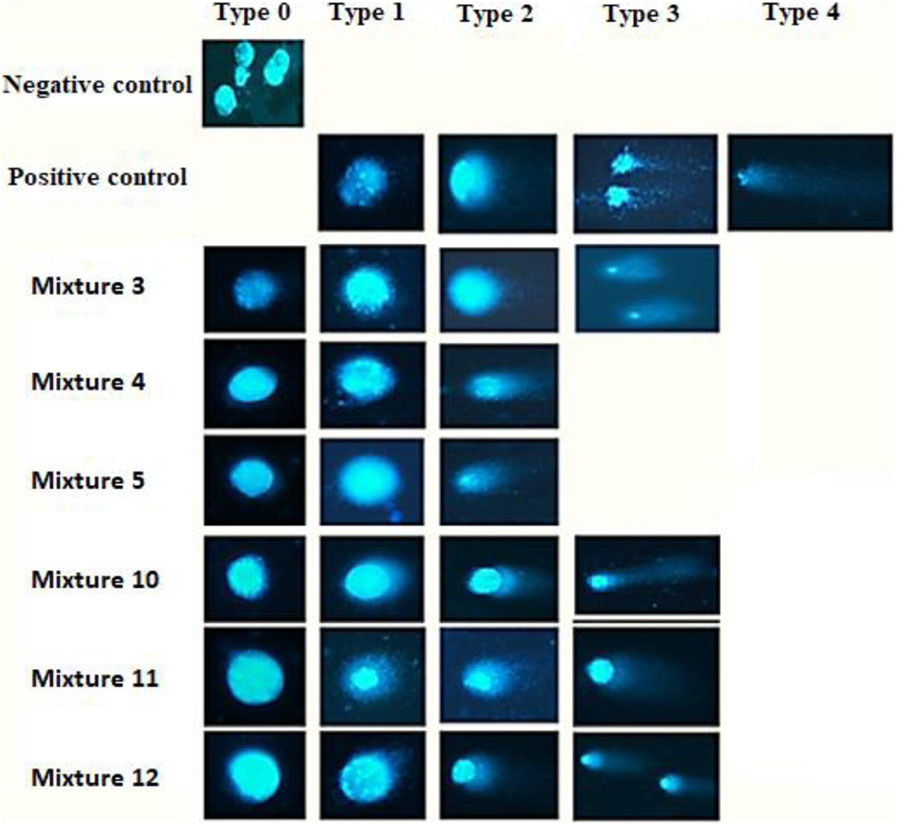
Genotoxicity observed by fluorescence

Electrophoresis was carried out in an alkaline medium to assess macrophage genome damage caused by the treatment compounds. Higher levels of damage were observed in cells treated with BZN (type 3 damage to the genome in 65% of the cells), compared to the ACT1 + BZN and ACT1 + ACT2 interactions. This DNA damage was significant reduced when ACT1 was used in combination with BZN (Mixture 10- 2X IC_50-ACT1_ + 1X IC_50-BZN_), where 49.7% of the cells had no damage (type 0), 54% had type 1 damage, and the remaining percentage exhibited type 2 damage. The least genotoxic therapy was found to be Mixture 4 (2X IC_50-ACT1_ + 1X IC_50-ACT2_), in which 57% of the cells did not present any type of damage, and 41% evidenced type 1 (Table 4 and Fig. 3). None of the fractions mixtures (ACT1 + ACT2) resulted in type 4 damage (maximum damage), which did, however, occur in 2.7% of cells treated with BZN.

**Table 4:** Damage levels observed on J774A.1 macrophages treated with a matrix of pharmacological interactions

Mixture	Damage Level										Damage index (DI)		% Total damage		*p*
	Type 0		Type 1		Type 2		Type 3		Type 4						
	%	SD	%	SD	%	SD	%	SD	%	SD	%	SD	%	SD	
BZN	0	0	1	0.2	7.3	1.2	89	1.7	2.7	1.3	293	3.1	98	0.01	Ref
3	36	3.1	53	3.5	8	1.5	3	1.3	0	0	79	7.5	45	0.02	0.0001
4	57	4.6	41	3.0	2	0.7	0	0	0	0	45	6.2	36	0.02	0.0001
5	26	2.6	71	1.5	3	0.5	0	0	0	0	77	4.4	44	0.01	0.0001
10	41	2.1	54	2.6	4	1.2	1	0.1	0	0	66	1.5	41	0.004	0.0001
11	10	4	49	2.1	34	2.0	7	2.5	0	0	137	7.6	59	0.02	0.0001
12	5.7	1.5	90	1.5	4	1.2	0.3	0	0	0	99	3.2	50	0.008	0.0001

*SD* standard deviation, *Mixtures 3, 4, and 5*: different combinations of ACT1 + ACT2 as described in Table 2; *Mixtures 10, 11, and 12*: different combinations of ACT1 + BNZ as described in Table 2; *ACT1:* limonene-enriched fraction from Carvone-chemotype of *Lippia alba*; *ACT2:* citral/caryophyllene oxide-enriched fraction from Carvone-chemotype of *Lippia alba*; *BZN* Benznidazole

### Morphological changes and oxidative stress on J774A.1 macrophages

**Fig. 4:**
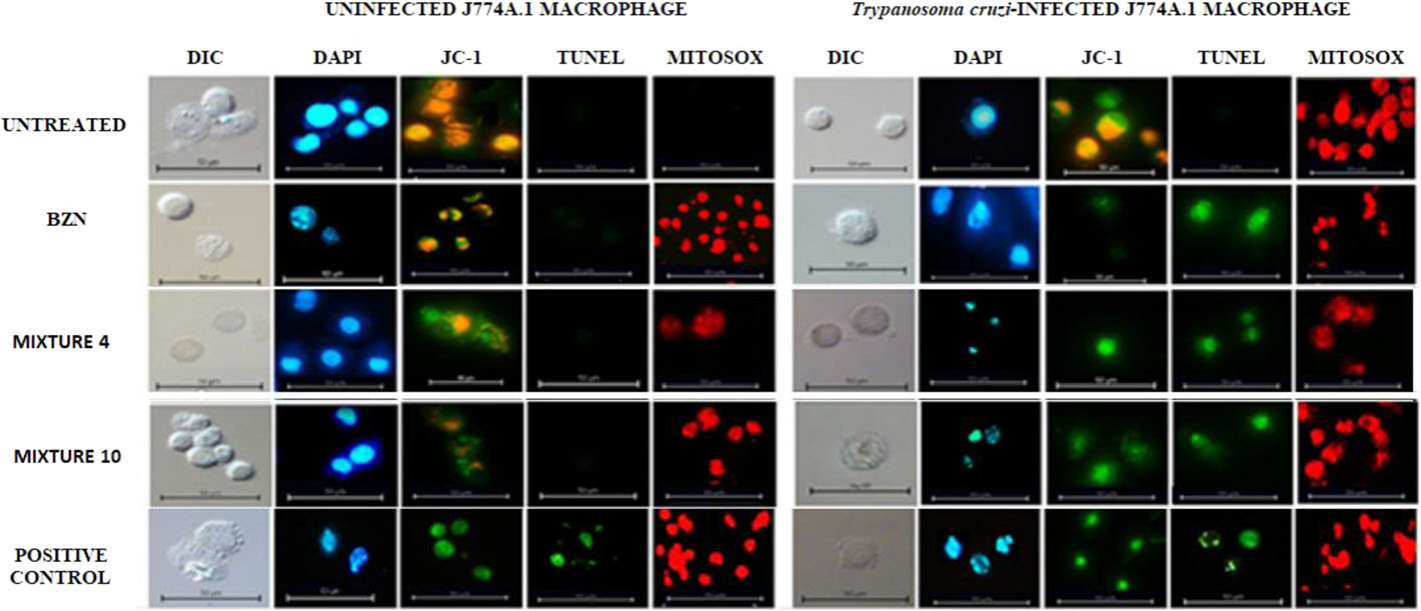
Morphological changes observed in J774A.1 macrophages treated with the best pharmacological interactions (representative fields)

Infected and uninfected macrophages were treated with the best pharmacological interaction yielded by the Mixture 4 of ACT1 + ACT2; and with ACT1 in combination with the BZN (Mixture 10). As shown in Fig. 4, uninfected macrophages, treated with essential oil fractions, exhibited a uniform morphology, without alterations at the level of the nucleus or mitochondria. Furthermore, the cells treated with the Mixture 10, despite showing a partial decrease in mitochondrial activity, maintained the stability of their genetic material. The infected and untreated macrophages did not show damage to their nuclei, but a slight decrease in mitochondrial activity was observed, possibly due to oxidative stress processes caused by *T. cruzi* infection. Conversely, when infected cells were treated with the Mixture 4 (Table 1), cell death was triggered in a significant percentage of the macrophages, likely by programmed cell mechanisms. This latter supposition stems from the fact that the characteristics of the cell death observed included chromatin fragmentation, nucleus condensation, and total loss of mitochondrial activity; as determined by Tunel, JC-1, and DAPI staining. Finally, the infected cells treated with the Mixture 10 (ACT1 + BZN) exhibited death due to both apoptotic-like (80%) and necrotic (20%) processes, with a total loss of mitochondrial activity.

**Fig. 5:**
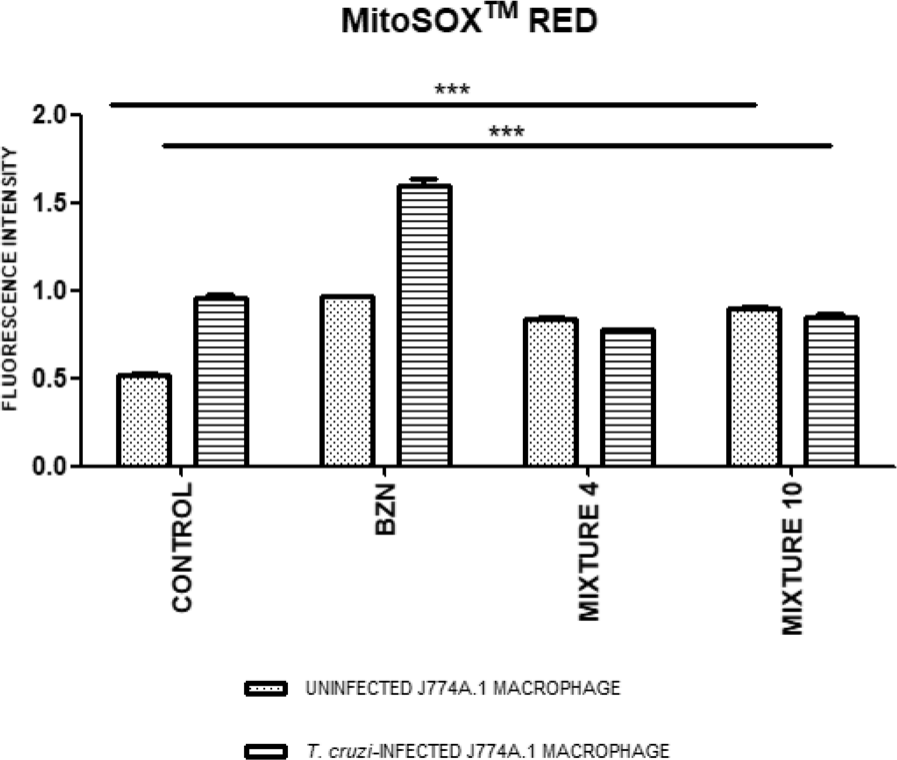
Fluorescence intensity data obtained by MitoSOX™ RED, *p < 0.05, **p < 0.01, ***p < 0.001

Additionally, the generation of mitochondrial ROS was evaluated using a MitoSOX™ Red probe. As illustrated in Figs. 4 and 5, macrophages infected with *T. cruzi* exhibited increased fluorescence, caused by the augmented ROS of the cell’s response mechanism to infection. Furthermore, it can be observed that stress is maintained in the case of the reference drug (positive control), possibly because BZN operates via the production of said ROS. In contrast, a significant decrease in fluorescence (*p* < 0.0001) was observed in macrophages infected and treated with the pharmacological interactions, Mixtures 4 and 10, likely due to an increase in antioxidant enzymes stimulated by the essential oil fractions. The fluorescence intensity data is detailed in Fig. 5.

### Bioenergy and mitochondrial function of J774A.1 macrophages infected with *T. cruzi*

For detection of OCR and ECAR, 80,000 cells/well were used as the optimal cell density, since this concentration provides adequate linear correlation in the equipment used. The Oligomycin and Rotenone/Antimycin inhibitors were used in the concentration recommended by the supplier, Seahorse Bioscience (Billerica, United States), which is 1 μM for both. The FCCP uncoupler concentration was established at 0.3 μM since, when using lower concentrations, the maximum oxygen consumption was not reached, and at higher concentrations, the rate of oxygen consumption was affected.

**Fig. 6:**
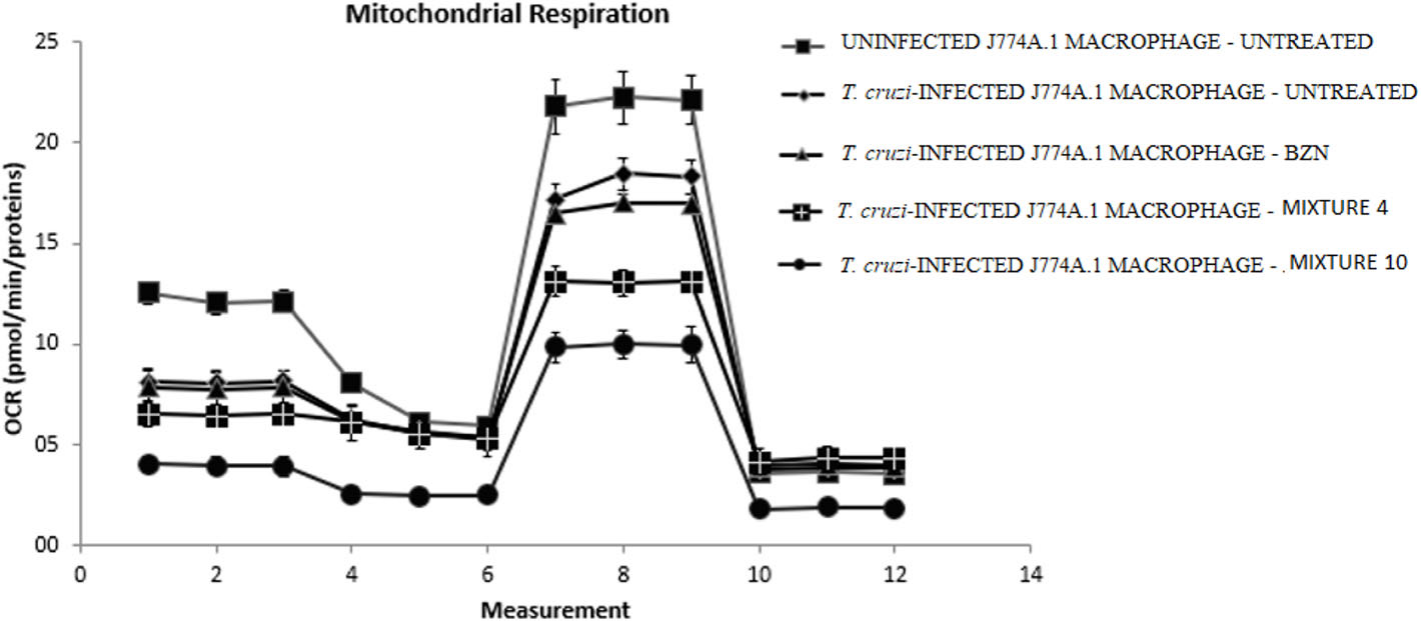
Effect on mitochondrial bioenergetics of J774A.1 macrophages subjected to an array of treatments

With respect to the effect on oxidative phosphorylation, infected macrophages showed a 6% decrease in maximum respiratory capacity compared to uninfected cells (Fig. 6). Likewise, the Mixtures 4 and 10 (applied in doses of CC_25_) generated a impair in the rate of oxygen consumption in infected macrophages, with this effect being even greater than that caused by the reference medicine evaluated individually (*p* < 0.05). Furthermore, both combinations showed a significant difference (*p* < 0.05) in all parameters, as compared to infected but untreated macrophages. With respect to the basal respiration rate, the Mixture 10 was observed to cause a 47% effect, while the Mixture 4 reduced it by 68%, in relation to those cells treated only with BZN (Fig. 6). As indicated in Fig. 4 and corroborated in Fig. 5, this mixture of ACT1 + ACT2 caused a decrease in membrane potential, most likely associated with proton leakage, which is statistically significant compared to the reference drug.

Similarly, it can be observed that the maximum respiratory capacity was reduced by 58% after treatment with the Mixture 4, and by 33% using the Mixture 10, compared to BZN, individually, and to those macrophages infected without treatment.

**Fig. 7:**
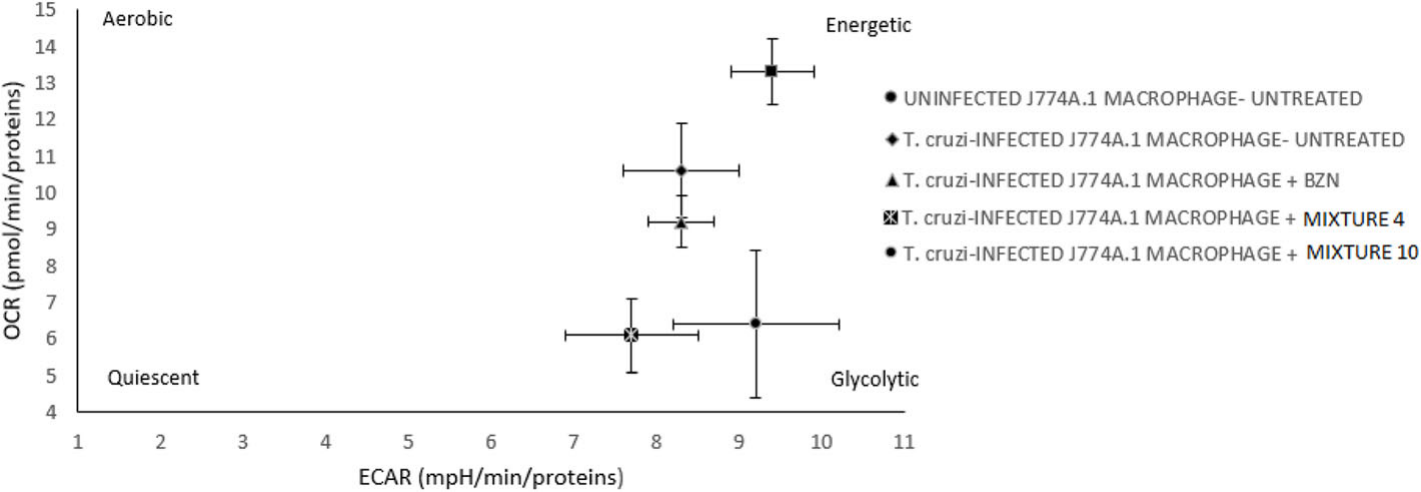
Energy map of infected and uninfected J774A.1 macrophages under an array of treatments

The energy map in Fig. 7 indicates that the uninfected model exhibits an essentially aerobic metabolism, with adequate functioning of the electron transport chain. However, in infected macrophages treated with BZN, a metabolic impairment is observed, reducing their energy capacity by 26%. This effect is significantly worsened by treatment using the Mixtures 4 and 10, with the infected macrophages exhibiting a mainly glycolytic metabolism and a > 50% reduction in their metabolic capacity.

### Modulation of the immune response

**Fig. 8:**
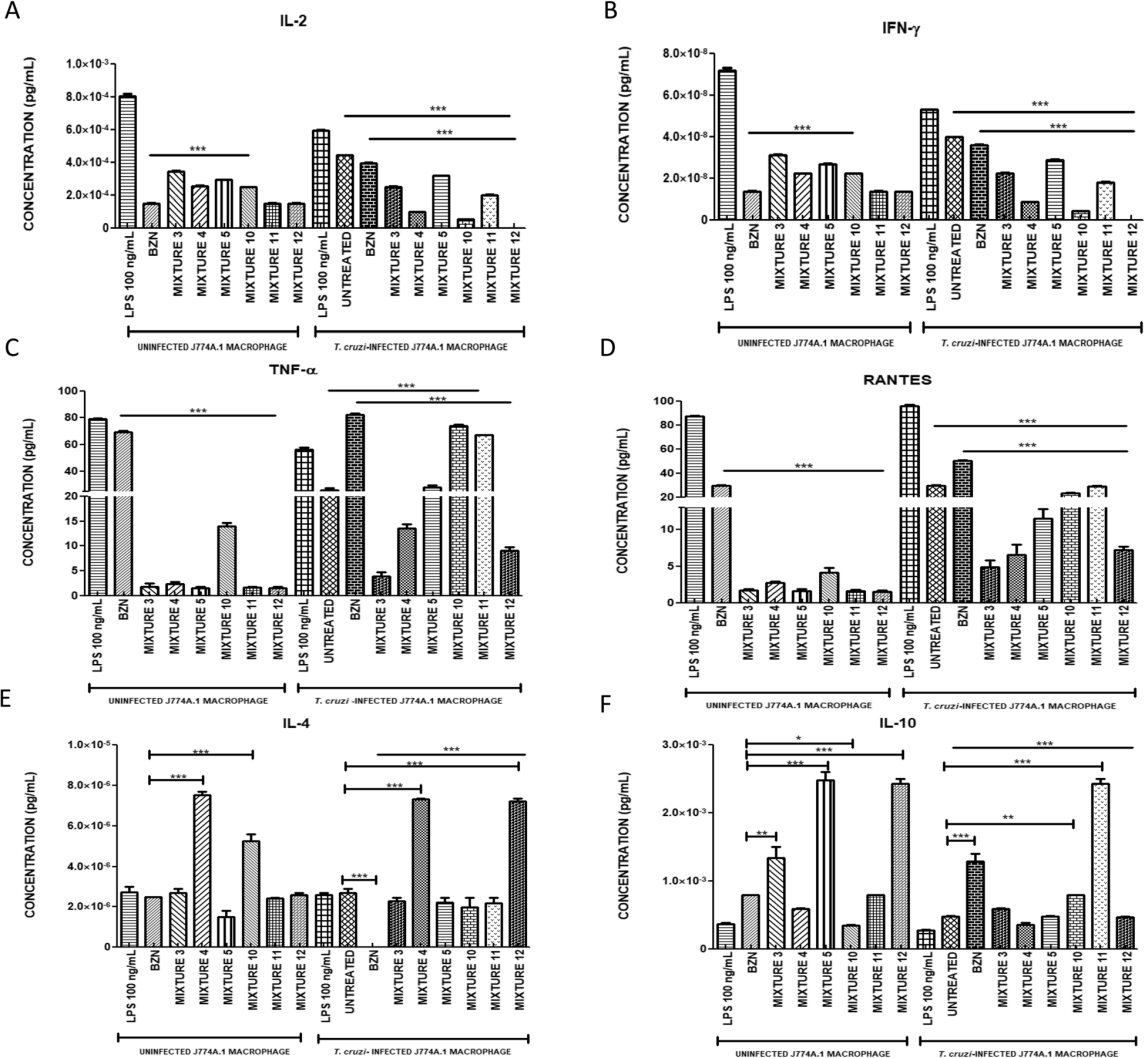
Pro- and anti-inflammatory cytokine levels of J774A.1 macrophages subjected to an array of treatments (LUMINEX)

Quantification of the pro- and anti-inflammatory cytokine levels was carried out by measuring them in the cell culture supernatant using Luminex technology. In this study, LPS was used as pro-inflammatory control for stimulation of uninfected and *T. cruzi-*infected macrophages. After LPS-stimulation, a consistent pro-inflammatory profile was observed in both cell models for all study cytokines (Fig. 8). As can be observed in Fig. 8, the readings obtained showed significant differences (*p* < 0.001) between the treatments using the mixtures, those using BZN, and the infected and untreated macrophages.

In general, the proinflammatory cytokines, IFN-γ and IL-2, exhibited a correlated behavior among therapies, in both cellular models. In the case of uninfected cells, the minimal values of these cytokines were observed after the BZN treatment, whether used alone (IFN-γ = 1.4 × 10^− 8^ pg/mL and IL-2 = 1.5 × 10^− 4^ pg/mL) or in combination with *L. alba* fractions (IFN-γ ≤ 2.9 × 10^− 8^ pg/mL and IL-2 ≤ 2.2 × 10^− 4^ pg/mL). Conversely, this medicine triggered the higher IFN-γ (3.5 × 10^− 8^ pg/mL) and IL-2 (3.9 × 10^− 4^ pg/mL) release in the culture supernatants by the infected cells. Nevertheless, these levels were significant reduced (*p <* 0.001) by the ACT1 interaction with BZN (IFN-γ ≤ 1.7 × 10^− 8^ pg/mL and IL-2 ≤ 1.9 × 10^− 4^ pg/mL). Consistently, all treatments based on fraction mixtures were able to decrease the IFN-γ and IL-2 levels, in infected macrophages. It should be noted that, in these cells, the greatest reduction in the values of the pro-inflammatory cytokines, IFN-γ and IL-2, was obtained by Mixture 12 (ACT1), which inhibited the production of both cytokines to below the detection limit of the test; while Mixture 4 (ACT1 + ACT2) caused a reduction of 76% compared to the levels observed in the BZN group. However, in all cases the production of IFN-γ was 5 times lower than that observed for IL-2 (Fig. 8a and b).

On the other hand, the levels of TNF-α and RANTES (also known as CCL5) produced were higher in macrophages infected with the parasite than in uninfected specimens. In the case of infected macrophages, this production was stimulated to a greater extent by the reference drug (BZN), making its levels higher in treated (TNF-α = 81.2 pg/mL and RANTES = 50.1 pg/mL) than in untreated (TNF-α = 70.3 pg/mL and RANTES = 30.3 pg/mL) macrophages (*p =* 0.001). Interestingly, the TNF-α and RANTES levels were significantly reduced (up to 98%) by the presence of ACT1 in the BZN-mixtures, especially in uninfected macrophages, which presented a minimal release of these two cytokines (TNF-α = 14.7 pg/mL and RANTES = 1.8 pg/mL for Mixture 10 and TNF-α = 1.7 pg/mL and RANTES = 1.5 pg/mL for Mixture 11) (Fig. 8c and d). Likewise, a significative decrease of these two cytokines were observed after therapies composed only by *L. alba* fractions, in both uninfected and infected cells. Note that, within the tested combinations of phytopharmaceuticals, Mixture 3 was that which resulted in the lowest levels of TNF-α and RANTES, with more than 94% reduction in non-infected macrophages and 95% in the infected by *T. cruzi*, compared to BNZ-group.

With respect to the anti-inflammatory cytokines, IL-4 levels were found to be very similar for infected and uninfected macrophages. In the BZN-treated and infected group, however, these fell below the lower detection range. On the other hand, the treatment using only ACT1 (Mixture 12), as well as the Mixture 4, caused a significant increase of IL-4 (8 × 10^− 6^ pg/mL and 7.4 × 10^− 6^ pg/mL, respectively) in comparison to the infected and untreated macrophages (2.4 × 10^− 6^ pg/mL) (Fig. 8e).

In the case of IL-10, in the non-infected cell model, treatments with ACT1 (whether alone or in combination with ACT2) exhibited significantly higher levels (up to 66%) than the group treated with BZN. In contrast, infected and treated macrophages with the fraction mixtures showed a significant reduction of these levels. In this same model, BZN caused an important increase of IL-10 values, which were doubled in its combination with ACT1 (Mixture 11) (Fig. 8f). Table 5 summarizes all results obtained.

**Table 5:** Effects on macrophages treated with enriched fractions of *Lippia alba* essential oils

Activity	Treatment					
	None	BZN	Mix 3	Mix 4	Mix 10	Mix 12
Uninfected J774A.1 macrophages						
CC_50_ (μg/mL)	–	298	–	–	–	–
FIC^a^	–	–	–	1.7	0.9	1
O_2_^−^ Mitochondrial^c^	0.53	0.97	1.8	–	0.91	–
ΔΨm^b^	N	N	0.85	N	N	N
Genome DNA^d^	N	F	N	N	N	N
Cellular membrane^e^	N	N	N	N	N	N
Death phenotype^g^	ND	ND	ND	ND	ND	ND
IFN-γ (10^−8^ pg/mL)^h^	–	1.4	0	2.2	2.3	1.4
IL-2 (10^−4^ pg/mL) ^h^	–	1.5	3.2	2.6	2.4	1.5
TNF-α (pg/mL) ^h^	–	70.3	3.4	2.8	14.7	1.8
RANTES (pg/mL) ^h^	–	30.3	2.5	2.9	4.8	1.7
IL-10 (10^−3^ pg/mL) ^h^	–	0.8	1.9	0.6	0.4	2.5
IL-4 (10^−6^ pg/mL) ^h^	–	2.4	1.5	7.3	4.9	2.5
Genotoxicity^f^			–			
*% Type 0*	*100*	*–*		*57*	*41*	*6*
*% Type 1*	*–*	*1*	*36*	*41*	*54*	*90*
*% Type 2*	*–*	*7*	*53*	*2*	*4*	*4*
*% Type 3*	*–*	*89*	*8*	*0*	*1*	*0*
*% Type 4*	*–*	*3*	*3*	*0*	*0*	*0*
*T. cruzi*-Infected J774A.1 macrophages						
IC_50_ (μg/mL)	–	48	–	–	–	1
FIC^a^	–	–	0.4	0.6	0.4	–
O_2_^−^ Mitochondrial^c^	0.98	1.56	0.77	–	0.82	–
ΔΨm^b^	↓	↓	↓↓↓	–	↓↓↓	–
Genome DNA^d^	N	F	F	–	F	–
Cellular membrane^e^	N	D	N	–	N	–
Death phenotype^g^ *% Type of Death*	N	Nec *60*	Apop *80*	–	Apop *80*	0
IFN-γ (10^−8^ pg/mL) ^h^	4.0	3.5	2.2	0.8	0.4	0
IL-2 (10^−4^ pg/mL) ^h^	4.4	3.9	2.5	0.9	0.5	8.4
TNF-α (pg/mL) ^h^	24.6	81.2	3.9	12.6	73.3	7.1
RANTES (pg/mL) ^h^	29.3	50.1	4.8	6.5	23.0	0.5
IL-10 (10^−3^ pg/mL) ^h^	0.5	1.3	0.6	0.4	0.8	7.3
IL-4 (10^−6^ pg/mL) ^h^	2.4	0	2.5	7.4	2.5	8

^a^*CIF* Fractional Inhibitory Concentration, ^b^*ΔΨm* Loss of mitochondrial membrane potential (JC-1), *Mix* Mixture, *N* normal, *F* fragmented, *ND* Not detected, *D* discontinue, *Nec* necrosis, *Apop* apoptosis. ^c^MitoSOX™ fluorescence intensity; ^d^DAPI; ^e^Phase Contrast Microscopy; ^f^COMET; ^g^TUNEL; ^h^Chemiluminescence (LUMINEX)

## Discussion

In the chronic phase of Chagas disease, dilated cardiomyopathy is considered the most serious clinical manifestation, the severity of which is dependent on multiple factors such as parasite strain, mononucleated cell infiltration, and the oxidative stress response (factors which greatly affect the structure and function of the mitochondrial respiratory chain proteins) [6]. During *T. cruzi* infection, ROS can cause tissue destruction either by toxic secretions from the parasite or by reactions mediated via the host’s immune system. In this regard, it has been suggested that both ROS and RNS formation occur due to the stimulation of inflammatory mediators, which in turn leads to an oxidative burst in phagocytic cells [5].

Although the complexity of the etiology of Chagas disease is well recognized, both current conventional treatments, BZN and NFX [6, 8], are essentially trypanocidal; and there is no clear clinical evidence for the benefits of these drugs in the late phases of the disease [6]. Previously, some approaches to anti-chagasic medications involves clinical studies with conventional antimycotic drugs as posaconazole, ketoconzale, and itraconazole have been performed, but with no significant anti-trypanocidal effects [17]. Alternatively, non-trypanocidal therapies with antioxidants and anti-inflammatory agents have shown promissory activity in ameliorate the cardiac injury, in experimental acute infection induced in murine model [17]. In recent years, interest in therapies that use natural products has grown, since they offer an important platform to develop multi-objective drugs with low toxicity. In this vein, enriched fractions with terpenes such as caryophyllene oxide, citral, and limonene have shown themselves to exhibit significant beneficial qualities, being trypanocidal, chemo-protective, oral-bioavailable, and low-cost agents [10–12, 18]. These phytotherapies could be used as co-adjuvants to the conventional trypanocidal treatments, enhancing their antiparasitic qualities, and possibly helping to reduce their adverse effects.

In the present study, the ACT1 and ACT2 fractions of the essential oils extracted from *L. alba* Carvone (96% of Limonene) and Citral (with 32.8% of citral and 7.6% of caryophyllene oxide) -chemotypes were obtained and studied in their in vitro trypanocidal, antioxidant and immunomodulatory effects on macrophages infected by *T. cruzi*. Both enriched fractions showed promising anti-proliferative effects on J774A.1 macrophages infected with *T. cruzi* amastigotes (IC_50 ACT1_ = 45 μg/mL and CC_50 ACT2_ = 80 μg/mL), with ACT1 being the most selective and least cytotoxic agent. By pharmacological interactions analysis, a noteworthy synergistic effect on amastigotes (trypanocidal) was observed for all the evaluated combinations (ACT1 + ACT2 and ACT1 + BZN); with an antagonistic effect (less toxic) on uninfected macrophages for ACT1 + ACT2; and an additive (more cytotoxic) action for ACT1 + BZN. Similarly, an improve in the anti-*T. cruzi* action was previously observed for interactions between limonene + caryophyllene oxide (∑CIF = 0.7), and between limonene and BZN (∑CIF = 0.6); with a impair of the toxicity on non-infected Vero cells by the combination of limonene with caryophyllene oxide (∑CIF = 1.2) [10]. Likewise, enhanced trypanocidal effect was also reported for the lupenone and caryophyllene oxide combination [19].

Morphological studies of the cell death phenotype suggested that the mixture of ACT1 and ACT2 induces a possible programmed cell death mechanism, which can be inferred due to the loss of cytoplasmic volume, the fragmentation of the nucleus (DAPI and Tunel), and the decrease in the mitochondrial membrane potential (JC-1), in both parasite and infected-macrophage cells. In contrast, BZN induced an increase in cytoplasmic volume and cell membrane discontinuity in most of the infected macrophages treated. It is important to mention that this effect was reversed using ACT1 in combination with BZN. These results are consistent with the literature, in which treatment with enriched fraction derived from *L. alba* triggered programmed cell death characteristics in cyclic forms of *T. cruzi* and Vero cells infected by the parasite [10].

To assess the therapies’ effect on oxidative stress, both infected and uninfected macrophages were studied via red MitoSOX™ probe, which specifically marks the mitochondrial superoxide ion. Through this marker, an increase in the intensity of fluorescence was detected in both infected and uninfected but treated models. This effect could be associated with the activation and production of ROS, as part of the mechanisms used by the macrophage either to counteract the invasion of the parasite or as a therapy response, respectively [20]. However, this increase was significantly higher in cells infected by *T. cruzi* and treated with BZN. This phenomenon can be associated with the trypanocidal mechanism of BZN, which is attributed to its ability to generate oxidative damage in the parasite [4]. In contrast, in infected macrophages treated with the best pharmacological interactions studied (Mixtures 4 and 10), a significant reduction in the levels of the mitochondrial superoxide ion was found in comparison to the reference therapy (BZN). This effect could be ascribed to the high percentages of limonene in ACT1 enriched fraction, considering that this terpene has previously been found to counteract oxygen free radicals and protect cells – and even organisms – against oxidative damage [21]. In this manner, a mitochondrial antioxidant effect of a trypanocidal therapy could be interesting, considering that treatment with BZN followed by a supplement of antioxidant vitamins such as E and C, reduces premature ventricular contractions, in patients with cardiac involvement from *T. cruzi* infection [22].

Note that, although the cells treated with the enriched fractions, ACT1 and ACT2, presented lower mitochondrial superoxide ion levels (compared to the BZN therapy), the oxidative stress determined by the MitoSOX™ staining was significantly inferior in the uninfected and untreated macrophages. Hence, the anti-proliferative mechanism of the ACT1 and ACT2, both on the parasite and on the infected macrophages, was apparently due to the induction of oxidative stress, in a similar trypanocidal effect to those described for BZN or NFX [6, 8], but with lower intensity. This effect may be attributable to an ROS elevation generated by the treatment with the fractions, but with a possible simultaneous induction of enzymatic antioxidant systems (previously described for limonene, in other cell models) [23]. This, in turn, may explain the selective anti-proliferative action previously reported for limonene on cells sensitive to oxidative stress, such as tumor cells or protozoa, in which there is exacerbated proliferation accompanied by a depletion of antioxidant defenses [10, 18].

To further investigate the effects of ACT1 and ACT2 on mitochondrial activity, the current study carried out cellular bioenergetics’ tests using Seahorse technology on infected macrophages. The Mixture 4 was found to induce a significant reduction in oxygen consumption rates (OCR), compared to macrophages without treatment or treated with BZN. Consistently, these essential oil fractions also stimulated a reduction in the basal oxygen consumption rate, maximal respiratory capacity, reserve respiratory capacity, and coupled respiration. These alterations suggest that both ACT1 and ACT2 therapies induce global damage to oxidative metabolism and, consequently, to ATP generation, which, over time, could lead to a cell death process, possibly by apoptosis. To the best of our knowledge, this is the first report evaluating the cellular respiration parameters of J774A.1 macrophages infected with amastigotes of *T. cruzi.*

The study also evaluated possible immunomodulating action by the compounds in question, considering that, patients in the chronic phase of the disease show an inflammatory response of greater intensity compared to those in the indeterminate phase of infection. In this regard, IFN-γ has been described as the central cytokine in the activation of *T. cruzi*-infected macrophages and cardiomyocytes, to produce TNF-α, NO, and ROS [6]. The current research found that in macrophages infected by *T. cruzi* and treated with BZN, there was an increase in all pro-inflammatory cytokines measured (IFN-γ, IL-2, RANTES, and TNF-α), without detectable production of IL-4 (anti-inflammatory). Interestingly, the use of ACT1 (whether alone or in combination with BZN or ACT2) had an immunomodulatory effect (reduction of all pro-inflammatory with significant elevation of IL-4). It is noteworthy, however, that this modulating action of the immune response favoring Th2/Th1 was more evident in the treatment of infected cells using ACT1 (Mixture 12).

Similarly, the combination of BZN with ACT1, in the Mixture 11, improved the expression of the IL-10 in the *T. cruzi*-infected macrophages. With respect to the interactions composed only by *L. alba* fractions, these mixtures did not exhibit a stimulatory effect on the production of this cytokine in the infected model. However, in uninfected macrophages, an apparently protective effect did occur due to an increase in this interleukin, caused by the therapeutic action of ACT1 (Mixture 12) and the Mixture 5.

These findings are compatible with previously described results, where in an infected model, treatment with individual schemes of terpenes such as limonene, citral, or caryophyllene oxide resulted in some immunomodulatory effects, with increases in IL-4, IL-10, or IL-27 (anti-inflammatory); and reduction in proinflammatory cytokines like IFN-γ, TNF-α, or IL-1β [23]. Likewise, these results are also in agreement with the studies performed by Baldissera et al. [9], which showed that treating rats infected by *Trypanosoma evansi* with limonene-rich oils decreased their levels of pro-inflammatory cytokines such as TNF-α and IFN-γ, and increased IL-10 levels. De Souza et al. [24]  also reported that oils rich in limonene significantly increased IL-10. It is also worth noting that, previous studies have found IL-10 to act as a potent inhibitor of IFN-γ and of Th1 cell differentiation; and a deficiency of this interleukin is accompanied by a greater release of TNF-α, IFN-γ, IL-12, and ROS [25]. In fact, IL-10 genetically deficient mice showed higher mortality due to a change in the Th1 profile after infection by *T. cruzi*, with increasing levels of TNF-α and IFN-γ in cardiac tissue [25]. This finding is important, since IFN-γ has been described as the main factor responsible for tissue damage in Chagas disease (dilated cardiomyopathy, in general) being, in turn, the most important target for the modulation of the immune response, with special relevance to the differential progression of the chronic phase of the disease.

To identify whether the tested enriched fractions were toxic to genetic material (representing a potential risk to human health), genotoxicity was assessed via Comet assays. The results indicated that the reference medicine was highly genotoxic, with macrophages treated only with BZN exhibiting a total damage ratio of 98%. This is consistent with previous findings using both Comet and micronucleus assays, in which BZN was described as a highly mutagenic agent, inducing DNA damage even at therapeutic doses, according to data derived from the serum of patients [26]. These results can be explained by the antiprotozoal action mechanism of BZN, which is based on the formation of short-lived anions (nitro radicals) and other compounds, including hydroxylamine, after reduction of the nitro group by the action of nitro reductases present in the parasite [6]. These intermediate species can interact with DNA, causing chain breaks, destabilization of the helices, and preventing DNA synthesis [26]. In contrast, the majority (57%) of cells treated with the ACT1 + ACT2 mixtures did not exhibit any genomic damage (type 0), 41% exhibited mild damage (type 1), and only a minimal percentage (2%) exhibited type 2 damage, with no evidence of type 3 or 4 damage. Interestingly, the inclusion of ACT1 in the Mixture 10, significantly decreased the DNA damage caused by BZN (41% of cells without any damage and 54% with minimal or type 1 damage). In this regard, López [27] referenced data on the genotoxicity of *Lippia alba* and its enriched fraction, concluding that the major compounds of this plant did not show genotoxic effects when tested on *Escherichia coli*. Likewise, limonene has also been found to reduce the toxicity of certain substances by interaction when mixed. One such case is that described by Nagpal [28], where limonene decreased by 60.8% the genotoxic effect of urethane, a strongly carcinogenic compound. Similarly, another study reported similar results for limonene in the genotoxicity of hydrogen peroxide [29]. Caryophyllene oxide, as well, does not induce mutations or chromosomal damage [30].

## Conclusions

This work presents enriched fractions (ACT1 and ACT2), derived from Citral and Carvone-chemotype essential oils of *L. alba*, as promising agents for the design of adjuvant therapies for *T. cruzi* infection. These fractions evidenced synergistic trypanocidal and immunomodulatory activities (ACT1 + ACT2 or ACT1 + BZN), that could benefit the anti-inflammatory/pro-inflammatory ratio, in the infected cell model. Additionally, these therapies showed very low toxicity (even counteract the toxic effects of BZN therapy) on non-infected cells, involving an apparently programmed cell death mechanism (non-inflammatory), with impairment of mitochondrial function and energy metabolism.

## Supplementary Information

**Additional file 1: Supplementary Figure.** Effects on cells treated with enriched fractions of *Lippia alba* essential oils*.*

## Data Availability

The datasets used and/or analyzed during the current study are available from the corresponding author on reasonable request.

## Abbreviations

μg: Microgram
μL: Microliter
μm: Micrometer
μM: Micromolar
ΔΨm: Loss of mitochondrial membrane potential
ΣFIC: Sum FIC
$\overline{\mathrm{X}}$ΣFIC: Sum average FIC
ACT1: Limonene-enriched fraction from Carvone-chemotype of *Lippia alba*
ACT2: Citral/caryophyllene oxide enriched fraction from Citral-chemotype of *Lippia alba*
ANOVA: Analysis of variance
Apop: Apoptosis
AT: Agilent Technologies
ATP: Adenosine − 5- triphosphate
BZN: Benznidazole
CD: Cluster of differentiation
CC_50_: Cytotoxic Concentration 50
CENIVAM: National research center for agroindustrialization of aromatic and medicinal tropical species
CO_2_: Carbon dioxide
DAPI: 4′,6-Diamidino-2-phenylindole
D-MEM: Dulbecco’s modified eagle medium
DMSO: Dimethylsulfoxide
DNA: Deoxyribonucleic acid
d-UTP: 2′-Deoxyuridine, 5′-triphosphate
ECAR: Extracellular acidification rates
EO: Essential Oil
eV: Electron volt
FCCP: Fluoro-carbonyl cyanide phenilhydrazone
FCV: Fundación cardiovascular de Colombia
FIC: Fractional inhibitory concentration
GC: Gas chromatography
GC/MS: Gas chromatography coupled to mass spectrometry
GPx: Glutathione peroxidase
GSH: Reduced glutathione
IC_50_: Inhibitory concentration 50
ICETEX: Instituto Colombiano de crédito educativo y estudios técnicos en el exterior
iFBS: Inactive fetal bovine serum
IFN-γ: Interferon gamma
IL: Interleukin
kg: Kilograms
*L. alba*: *Lippia alba*
LIT: Liver infusion tryptose
LPS: Lipopolysaccharide
m^3^: Cubic meter
min: Minute
Mix: Mixture
mL: Milliliter
mm: Millimeter
MN-SOD: Manganese-dependent superoxide dismutase
ND: Not detected
Nec: Necrosis
NFX: Nifurtimox
nm: Nanometer
nM: Nanomolar
NO: Nitric oxide
OD: Optical density
OCN: Caryophyllene oxide
OCR: Oxygen consumption rates
PBS: Phosphate buffered saline
pg: Picogram
RNS: Reactive nitrogen species
ROS: Reactive oxygen species
SD: Standard deviation
SI: Selectivity index
*T. cruzi*: *Trypanosoma cruzi*
TcI: *Trypanosoma cruzi* I
TdT: Terminal desoxynucleotidyl transferase
TNF: α Tumor necrosis factor alpha
UDES: Universidad de Santander
USA: United States of America
USD: United States dollars
