# Supplementary figures and images for: Immunomodulatory, trypanocide, and antioxidant properties of essential oil fractions of Lippia alba (Verbenaceae)
Source: BMC Complement Med Ther. 2021 Jul 2;21:187. doi: 10.1186/s12906-021-03347-6 (PMC8254251; doi:10.1186/s12906-021-03347-6)

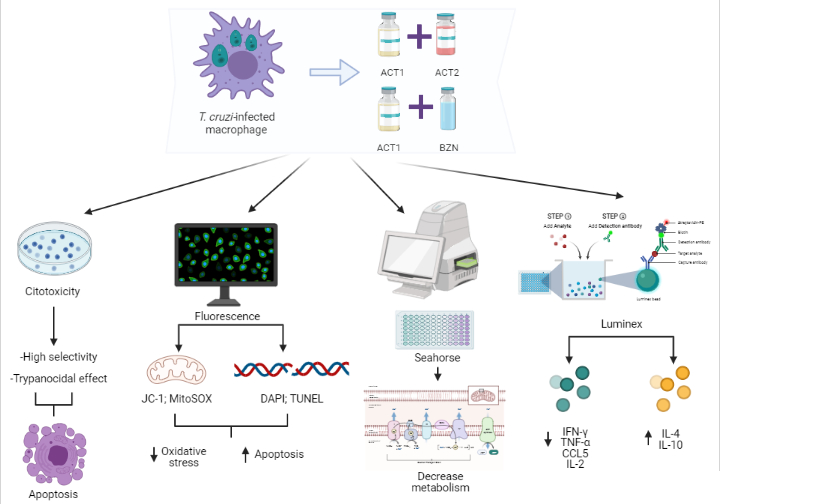

Supplement: Supplementary file 1 — Additional file 1: Supplementary Figure. Effects on cells treated with enriched fractions of Lippia alba essential oils. [file 12906_2021_3347_MOESM1_ESM.tif]
